# Video Multiple Watermarking Technique Based on Image Interlacing Using DWT

**DOI:** 10.1155/2014/634828

**Published:** 2014-12-21

**Authors:** Mohamed M. Ibrahim, Neamat S. Abdel Kader, M. Zorkany

**Affiliations:** ^1^National Telecommunications Institute, Cairo 11678, Egypt; ^2^Faculty of Engineering, Cairo University, Cairo 12316, Egypt

## Abstract

Digital watermarking is one of the important techniques to secure digital media files in the domains of data authentication and copyright protection. In the nonblind watermarking systems, the need of the original host file in the watermark recovery operation makes an overhead over the system resources, doubles memory capacity, and doubles communications bandwidth. In this paper, a robust video multiple watermarking technique is proposed to solve this problem. This technique is based on image interlacing. In this technique, three-level discrete wavelet transform (DWT) is used as a watermark embedding/extracting domain, Arnold transform is used as a watermark encryption/decryption method, and different types of media (gray image, color image, and video) are used as watermarks. The robustness of this technique is tested by applying different types of attacks such as: geometric, noising, format-compression, and image-processing attacks. The simulation results show the effectiveness and good performance of the proposed technique in saving system resources, memory capacity, and communications bandwidth.

## 1. Introduction

Due to the rapid growth of computer and network technologies, digital multimedia are becoming more popular and it is very easy to transmit and distribute data. So, there is a demand for techniques to protect the data and prevent unauthorized duplication. Digital watermarking protects the illegal copying of multimedia. A watermark is secret information about origin, ownership, copy control, and so forth. This information is embedded in multimedia content, taking care of robustness and imperceptibly. The watermark is embedded and extracted as per requirement to represent the ownership and/or the identity of multimedia [1].

Digital media files may be text, images, audios, or videos. So, digital watermarking is classified into four types: text watermarking, image watermarking, audio watermarking, and video watermarking. The watermark may be in a noise form such as Pseudo-random Gaussian noise, or in a data form such as text, image (logo), and video. Also, digital watermarking is classified according to the number of watermarks per the host media file into two types: single and multiple watermarking.

**Figure 1:**
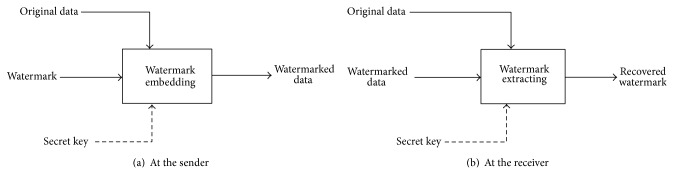
Classical nonblind watermarking system.

Digital watermarking systems are also categorized according to the watermark extracting process into two types: nonblind and blind watermarking [2]. In the nonblind watermarking systems, as shown in Figure 1, the original media is required in addition to the watermarked media in the watermark extracting process, while in blind watermarking the original media is not required in the watermark extracting process [3]. From this definition, the two advantages in nonblind watermarking techniques over the blind ones are as follows: first, being lower in computational complexity and second more robustness against attacks or signal distortions because the extracted watermark is more similar to the original one. But also this definition presents the main problem of the nonblind watermarking systems: double overhead over system resources like memory/storage in both sender and receiver and bandwidth on the communications channel between them.

Digital video can be defined simply as a collection of sequential images [3]. So, video watermarking can be considered as an expansion of image watermarking. Video watermarking system has more capacity than image watermarking system, so more watermarks that included images and videos can be embedded in the video watermarking system (as proposed in this paper). The previous problems of the nonblind watermarking have a more effect on video watermarking systems. So, a solution for this problem is a more important issue in video watermarking.

A proposed image watermarking technique based on image interlacing has been used to solve the memory capacity and communications bandwidth problems of the nonblind watermarking systems [4]; the target system was image watermarking system with a single watermark (color image as host and gray image as watermark). In this paper, video watermarking and multiple watermarks in different types (gray image, color image, and video) have been introduced.

This paper is organized as follows. A brief review of video watermarking techniques is presented in Section 2. The based techniques which the proposed method depends on are illustrated in Section 3. The proposed video multiple watermarking technique is presented in Section 4. Simulation results and discussions are given in Section 5 and finally the conclusion and the references are included.

## 2. Video Watermarking Techniques

Digital video is a sequence of still images called frames and these frames are loaded by a constant rate called frame rate. So, by reading the frames from the video file frame-by-frame and dealing with each frame as a color image, all image watermarking techniques can be used as video watermarking techniques [5].

As in image watermarking, the watermark embedding and extraction operations can be done in spatial domain or frequency domain. In the spatial domain, the watermark is embedded by modifying the pixel values of the host image/video directly. In the frequency or transform domain, the watermark is embedded by modifying the frequency components of the host image/video [5]; this is done by transforming the host signal to the frequency domain before embedding the watermark and retransforming to spatial domain after that. As a result, spatial domain watermarking techniques are lower in complexity than frequency domain techniques. The characteristics of the human visual system (HVS) are better captured by the frequency coefficients than spatial coefficients [1]; thus, frequency domain watermarking techniques enjoy better imperceptibility, more robustness against attacks such as noise addition, pixel removal, rescaling, rotation, and shearing, plus more compatibility with compression standards such as MPEG 1, 2, and 4 (Moving Picture Experts Group).

Unlike image watermarking, there is a third type of watermarking techniques called compressed-domain video watermarking techniques [6]. Many digital videos are typically stored and distributed in compressed form (e.g., MPEG). Due to the real-time requirements of video broadcasting, there is no time for decompression and recompression, so the watermark in these techniques is embedded directly in the compressed video. The main disadvantage of these techniques is that the watermarked video can be highly susceptible to be recompressed with different parameters or converted to formats other than MPEG.

## 3. The Based Techniques

The proposed technique in this paper depends on image interlacing. When deploying this technique to the video multiple watermarking system, three-level discrete wavelet transform (DWT) is used as an example of the frequency domain watermarking. For more security, Arnold Transform as one of the popular image/video frame encryption methods is used to encrypt the watermarks before embedding them in the host video. The following subsections will discuss these techniques before starting with the proposed method.

### 3.1. Three-Level DWT

Discrete fourier transform (DFT), discrete cosine transform (DCT), and discrete wavelet transform (DWT) are the most popular reversible transforms used in frequency domain watermarking. But DWT as a multiresolution multilevel transform is much preferred in watermarking than DCT and DFT, because it understands the human visual system (HVS) closer than them [7].

**Figure 2:**
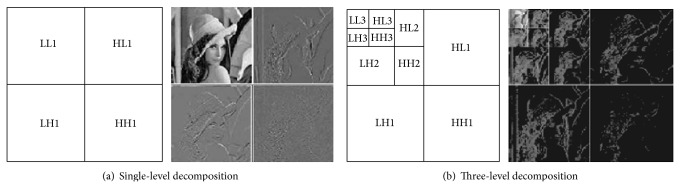
One- and three-level DWT decomposition of Lena image.

At the first level of DWT decomposition, the image/video frame is filtered horizontally and vertically by low (L) and high (H) pass filters to produce four frequency subbands [5]: LL1 as the low frequency subband and HL1, LH1, and HH1 as the high frequency subbands. The low frequency components contain the most significant portions of the image/video frame in which any modifications like watermarking can damage it [8], as shown in Figure 2(a) [9]. The high frequency components contain the least significant portions of the image/video frame which can be eliminated by compression. So, the mid frequency components are the best locations for watermarking. These mid frequency components appeared strongly at the next levels of DWT decomposition in which the LL (*n*) subband is decomposed into LL (*n* + 1), LH (*n* + 1), HL (*n* + 1), and HH (*n* + 1), where “*n*” is the level number, LL (*n* + 1) is the lower frequency subband, and LH (*n* + 1), HL (*n* + 1), and HH (*n* + 1) are the mid frequency subbands [8].

Three-level DWT as shown in Figure 2(b) [10] is used in the proposed method. This number of decomposition levels is enough to present the mid frequency components strongly such as the HL3 subband which is considered as the best location for watermarking operations according to the HVS properties [8]. So, in our case, more than three levels of decomposition is unnecessary, as well as it will be more computational complexity.

### 3.2. Arnold Transform

In order to enhance the security and robustness of the watermarking system, the watermark image/video frame is encrypted before embedding. Arnold Transform is one of the popular image/video frame encryption methods [4, 11]. In this transform, which is a periodic transform, the original organization of the image/video frame pixels is randomized, and after a number of iterations called Arnold's period the image/video frame is returned to its original state. Arnold Transform uses the following formula for changing the location of each pixel in the image/video frame of size *N* × *N*:

(1) $$\begin{array}{c} \left(\begin{array}{c} x^{\prime }\\ y^{\prime } \end{array}\right)=\left(\begin{array}{cc} 1 & 1\\ 1 & 2 \end{array}\right)\left(\begin{array}{c} x\\ y \end{array}\right)\;\left(\mathrm{mod}\;\;N\right), \end{array}$$

where (*x*, *y*) is the current location and (*x*′, *y*′) is the new location.

**Figure 3:**
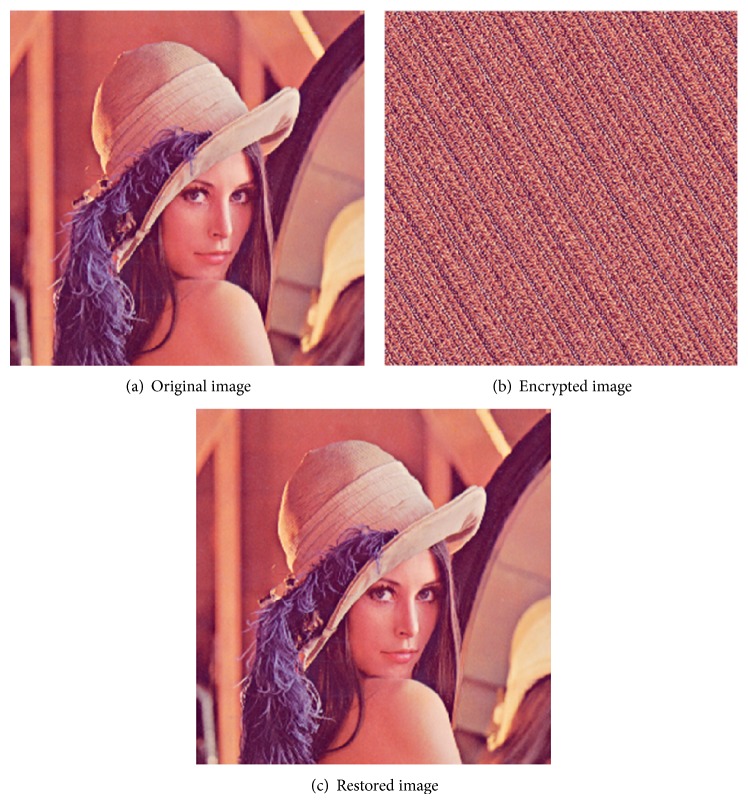
Arnold Transform as image/video frame encryption method.

To use the Arnold Transform as an encryption method of type symmetric, a number of iterations which must be less than the Arnold period can be used as a symmetric encryption key [12].  Figure 3 shows the encryption of “Lena” image using Arnold Transform.

### 3.3. Image Interlacing

Image interlacing (also known as interleaving) is an operation in which any image can be divided into subimages. Deinterlacing is the reverse operation in which the subimages are combined together to generate the original image. The interlacing algorithm indicates the contents of each subimage, and in the proposed method the interlacing algorithm is depending on dividing the image rows into even and odd rows and the image columns into even and odd columns [4].

In the proposed method, there are two levels of interlacing: one-level interlacing and two-level interlacing. One-level Interlacing has two types: interlacing by rows only to get even rows (ER) and odd rows (OR) subimages, as shown in Figure 8, and interlacing by columns only to get even columns (EC) and odd columns (OC) subimages, as shown in Figure 9.

Two-level interlacing is as follows: interlacing by rows first and then by columns (or in the opposite order) to get four subimages: even rows even columns (EE), even rows odd columns (EO), odd rows even columns (OE), and odd rows odd columns (OO) as shown in Figure 10.

## 4. Proposed Technique

In the nonblind video watermarking system as shown in Figure 1, two identical copies of the original video are used. At the sender, one of them is watermarked and then is sent to the receiver. The other copy of the original video is sent as it is. At the receiver, the watermark is recovered using both the watermarked video and the other copy of the original video.

The paper goal is how to prevent sending this original video to the receiver to save the system resources like memory, storage, and communications bandwidth. The idea of our proposed solution for this problem is as follows: if there is a technique by which the original video is divided into parts or subvideos, from these subvideos we can get two of them that are identical (or at least are very similar to each other). These two subvideos can play the same role of the two identical copies of the original video, in such case as a result, there is no need to another copy of the original video in the watermark extracting operation at the receiver which is the goal of this paper.

This technique is the image interlacing for image watermarking, and the same technique can be used in videos but in two steps: first, the video frames are divided into subframes using image interlacing and then these subframes are collected together to generate the subvideos.

The proposed watermark embedding and extracting processes are passing through the following steps.

### 4.1. At the Sender (The Watermark Embedding Process)

**Figure 4:**
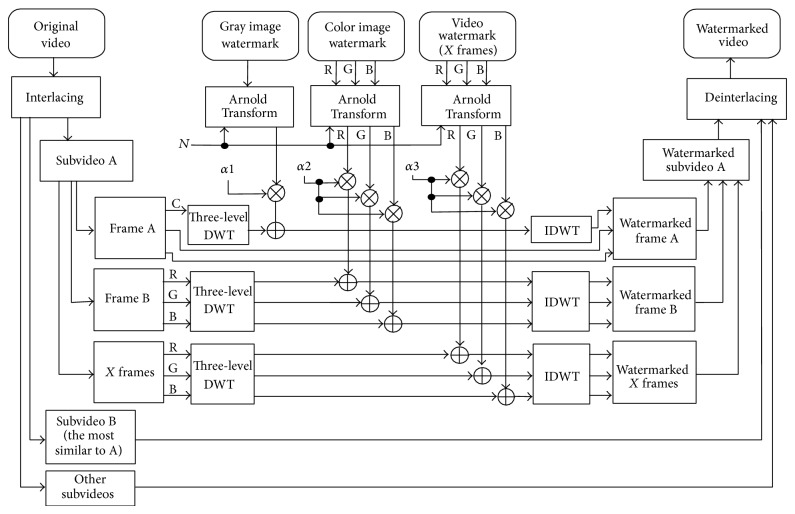
The watermark embedding process in the proposed video multiple watermarking system, where RGB stands for red, green, and blue color bands,* N* is the number of Arnold Transform iterations, *α* is the scaling factor, C is the selected color band, A and B stand for the most similar subvideos, and DWT stands for discrete wavelet transform.

The proposed watermarking technique main structure at the sender is given in Figure 4.

*First Step*. The original video is interlaced into subvideos.

**Figure 5:**
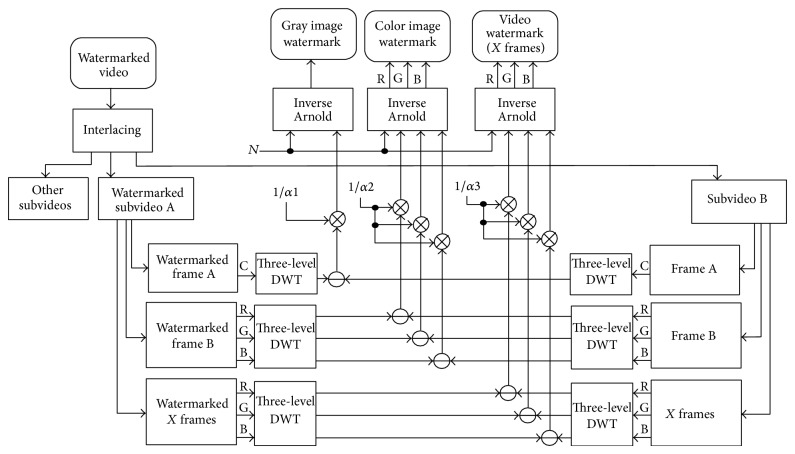
The watermark extracting process in the proposed video multiple-watermarking system.

*Second Step*. The most similar two subvideos (i.e., subvideos A and B as shown in Figures 4 and 5) are selected by calculating the similarity factor or normalized correlation (NC) between the two subvideos. This calculation of NC is performed first between each of the corresponding two color bands in each corresponding two subframes using the following formula [13]:

(2) $$\begin{array}{c} \text{NC}=\frac{1}{w\times h}\overset{w}{\underset{i=1\;}{\sum }}\overset{h}{\underset{j=1}{\sum }}\overset{-}{\left(X_{i\cdot j}⊕X_{i\cdot j}^{\prime }\right)}, \end{array}$$

where *X*_*i*·*j*_ and *X*_*i*·*j*_′ are the corresponding pixel values in two subframes and (*w* × *h*) is the subframe size. Then, we calculated the average NC value for all color bands and for all subvideo frames. The two subvideos that give the biggest average NC value are the most similar two subvideos that resulted from the interlacing operation on the original host video. The names or numbers of these two subvideos will be the first part of the secret key between the sender and the receiver.

In the proposed method, the NC is used in two positions: first, in the second step to get the most similar two subvideos, and second, after the watermark extraction process to evaluate it by comparing the extracted watermark with the original one.

*Third Step*. Multiple watermarks in different types (gray image and/or color image and/or video) are embedded in the first one of the selected subvideos (Subvideo A). Each of them is embedded in separate frames, which means that the proposed method is not affected if one of them is not available and this is one of the strength points of our proposed method. The watermarked frame numbers corresponding to each watermark will be the second part of the secret key between the sender and the receiver.

Before the watermark embedding process, all watermarks are encrypted using Arnold Transform, by a specific number of iterations (*N* as shown in Figures 4 and 5), and this number will be the third part of the secret key between the sender and the receiver.

The watermark embedding process is performed after transforming the selected frames into frequency domain using three-level DWT and the following equation [4, 14]:

(3) I′=I+αW,

where *I* is the HL3 subband of original subvideo frame, *I*′ is the watermarked one, *W* is the watermark signal, and *α* is the scaling factor. This scaling factor is a constant value for all subvideo frames and the selection of this value is based on rules as will be presented later. Each watermark has its own scaling factor as shown in Figures 4 and 5 (*α*1, *α*2, and *α*3 for the three used watermarks). *α* for video made for every frame has been chosen and then the average value to represent the *α* value was calculated. These scaling factors will be the fourth and last part of the secret key between the sender and the receiver.

*Final Step*. The watermarked video is generated by deinterlacing the watermarked subvideo and other subvideos. This watermarked video and the secret key (of four parts) are sent to the receiver and no copy of the original video is sent (the paper goal).

### 4.2. At the Receiver (The Watermark Extracting Process)

The proposed watermarking technique main structure at the receiver is given in Figure 5.

*First*, the watermarked video is interlaced by the same level and type as the original video in the sender.* Second*. The watermarked subvideo A and the subvideo B were selected by using the first part of the secret key. Finally, the watermark extraction process is performed using the two selected subvideos as follows: first, select the frames from both selected subvideos using the third part of the secret key. Second, transform these frames into frequency domain using three-level DWT. Third, extract the watermarks using the watermark extraction equation which is expressed as [4, 14]:

(4) $$\begin{array}{c} W^{\prime }=\frac{\left(I^{\prime \prime }-I^{*}\right)}{\alpha }, \end{array}$$

where *I*′′ is the HL3 subband of the watermarked subvideo A frame, *I* ^*^ is the corresponding HL3 subband of the subvideo B frame, *W*′ is the extracted encrypted watermark, and *α* is the scaling factor from the fourth part of the secret key. Then, the extracted encrypted watermark is decrypted using inverse Arnold Transform by number of iterations from the second part of the secret key.

Let us talk in detail about the watermark embedding and extracting equations (3) and (4). For the grayscale image watermark, only one color band from one video frame is needed for watermark embedding and extracting operations. This color band (C as shown in Figures 4 and 5) is the color band that gives the best NC values when selecting subvideos A and B (the second step). For the color image watermark, all color bands from one video frame is needed, and each color band from this watermark is embedded into the corresponding color band from this video frame [15]. For video watermark, all color bands from the same number of frames in the video watermark are required, which means that the number of frames in the video watermark must be less than or equal to the number of frames in host video.

## 5. Simulation Results

**Figure 6:**
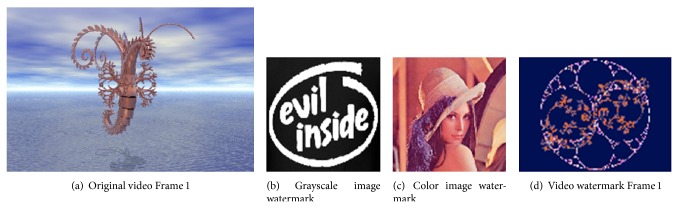
Host video and watermarks.

The proposed method is tested using multiple testing videos as host videos and multiple testing images and videos as watermarks.  Figure 6 presents one example of these host videos and examples of each watermark type as follows: “Airhorse.avi” as a host video of 50 frames with frame size 512 × 704 and frame rate 30 frames/second, “Evil Inside.jpg” as a grayscale image watermark of size 32 × 32, “Lena.jpg” as a color image watermark of size 32 × 32, and “Composite.avi” as a video watermark of 22 frames with frame size 32 × 32 and frame rate 15 frames/second.

There are many operations in the proposed method; each operation has its own results as follows.

### 5.1. Interlacing Operation

**Figure 7:**
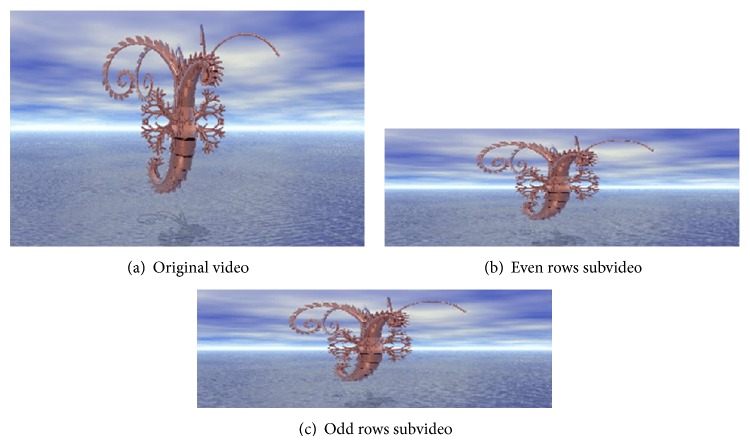
One-level interlacing by rows.

**Figure 8:**
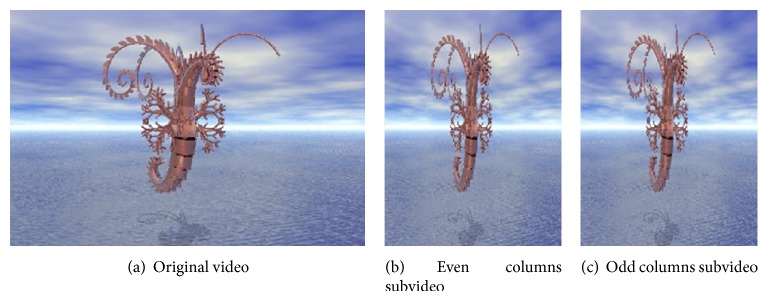
One-level interlacing by columns.

**Figure 9:**
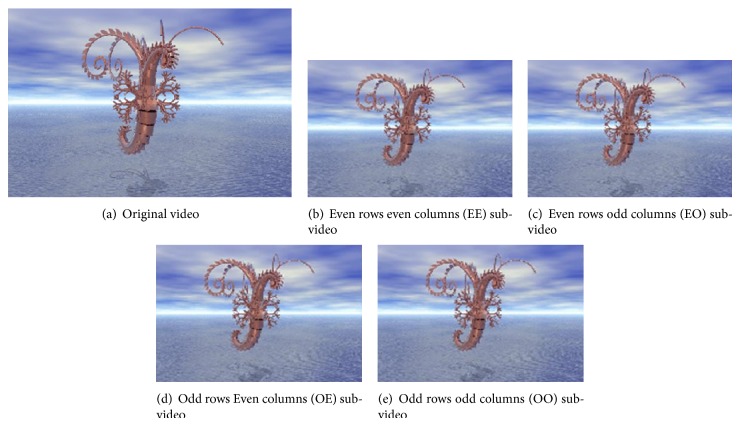
Two-level interlacing.

As in the proposed method, there are two levels of interlacing. Figures 7 and 8 present the results of the two types of one-level interlacing and Figure 9 presents that of the two-level interlacing. In these figures, the first frames from the original video and from each resulted subvideo are presented.

### 5.2. Selection of Subvideos A and B and the Color Band C

After the interlacing operation, we calculate the normalized correlation (NC, see (2)) between the two resulted subvideos to get the most similar two subvideos (subvideos A and B as shown in Figures 4 and 5) in which the watermarking operations will be performed later. Also, the color band that gives the best NC values will be the color band (C) from the specified frame (a) which is used for embedding and extracting the grayscale image watermark.

Table 1 shows the NC values between the two resulted subvideos in each interlacing level. From these results, we note that all NC values are close to one, which means that the used interlacing algorithm gives subvideos that are similar to each other. But if we compare between the two subvideos to get the most similar pair they will be EC and OC in one-level interlacing and OE and OO in two-level interlacing. If we compare between the color bands to get the one which have the best results, it will be the green.

### 5.3. Watermarking Operations

To show the results of our proposal, host video and watermarks in Figure 6 are illustrated as examples. The video watermark is embedded in the first 22 frames of the host video (*X* = 22), the grayscale image watermark is embedded in the green color band (from Table 1) of frame number 30 (a = 30), and the color image watermark is embedded in frame number 40 (b = 40). All of these watermarks are encrypted before embedding by number of iterations less than the Arnold Period as illustrated in Section 3.2. For 32 × 32 watermark image or video frames the Arnold Period is equal to 48 [11], so we can encrypt them by a number of iterations such as *N* = 20.

Two main tools are used for evaluating the watermarking systems: NC (normalized correlation) for evaluating the watermark extracting process by comparing the extracted watermark with the original one and PSNR (peak signal to noise ratio) [13] for evaluating the watermark embedding process by comparing the watermarked frame with the original one. For both NC and PSNR, we calculate the average value for all frames. In Table 1, NC values are the average values for all frames. In Table 2, both NC and PSNR values are the average values for all frames. In Table 3, both NC and PSNR values are for individuals' frames.

PSNR is defined in terms of mean square error (MSE) as follows:

(5) $$\begin{array}{c} \text{PSNR}=10\times \log_{10}\frac{255^{2}}{\text{MSE}},\\ \text{MSE}=\frac{1}{r\times c}\overset{r}{\underset{i=1\;}{\sum }}\overset{c}{\underset{j=1}{\sum }}{\left(H_{i\cdot j}⊕H_{i\cdot j}^{\prime }\right)}^{2}, \end{array}$$

where *H*_*i*·*j*_ and *H*_*i*·*j*_′ are the corresponding pixel values in the original and watermarked frames, respectively, and the size of each frame is *r* × *c*.

Tables 2 and 3 show the effectiveness and good performance of the proposed multiple-video watermarking technique.

### 5.4. Selection Scaling Factor Value *α*

**Figure 10:**
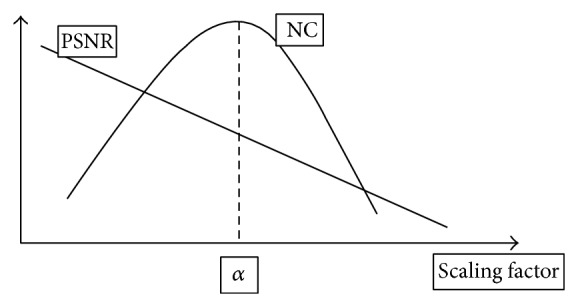
Scaling factor versus PSNR and NC.

The scaling factor *α* is the value that gives the best results in the evaluation of the watermarking system. There are two main parameters for evaluating such systems: PSNR and NC. As shown in Figure 10, there is a linear relationship between PSNR and scaling factor, the PSNR is reverse proportional to the scaling factor, the peak value of PSNR is when the scaling factor is equal to zero (No watermarking), so PSNR cannot be used as a reference for the best scaling factor value alone. But for NC, there is a nonlinear or Gaussian relationship between NC and scaling factor with a peak value, so the scaling factor *α* can be selected as the scaling factor value that gives the best NC value when evaluating the watermark extraction process. In this proposed paper, choice of *α* for video was made for every frame and then the average value to represent *α* value was calculated.

### 5.5. Robustness against Attacks Evaluation

During the communications channel between the sender and the receiver, the watermarked video is attacked; these attacks are categorized into five categories. These categories are as follows: geometric attacks, noising attacks, denoising (filtering) attacks, format-compression attacks, and image-processing attacks. In order to ensure that our proposed solution is not affected against the robustness of the video multiple watermarking systems against attacks, one or more examples from each category are applied on these systems before and after using the proposed solution as shown in the next subsection.

### 5.6. Comparison between the Classical Video Multiple Watermarking System and the Proposed System

**Figure 11:**
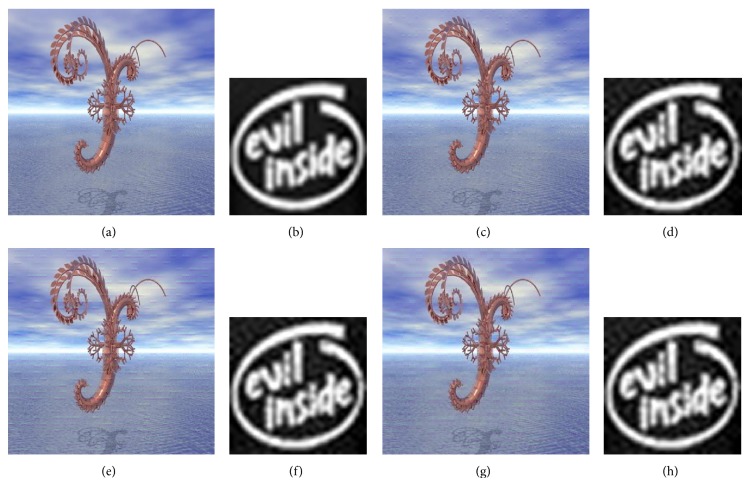
Comparison between the classical nonblind video multiple watermarking system (grayscale image watermark) and the proposed method where (a) and (b) show original video frame and original watermark, (c) and (d) show watermarked video frame and recovered watermark without interlacing, (e) and (f) show watermarked video frame and recovered watermark with one-level interlacing, and (g) and (h) show watermarked video frame and recovered watermark with two-level interlacing.

**Figure 12:**
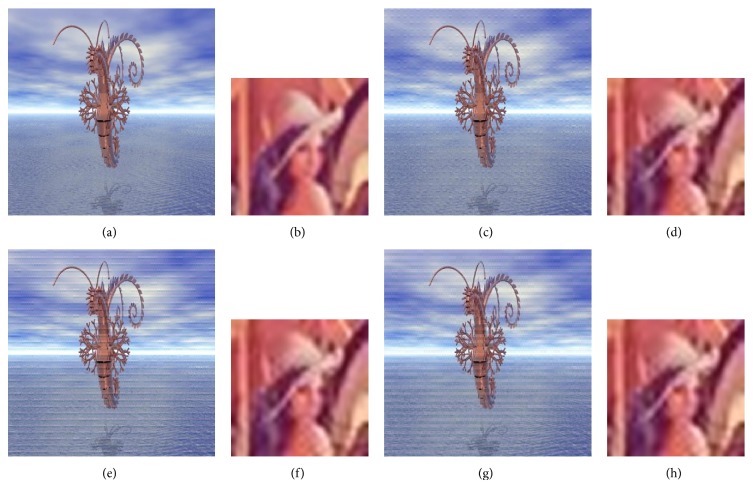
Comparison between the classical nonblind video multiple watermarking system (color image watermark) and the proposed method where (a) and (b) show original video frame and original watermark, (c) and (d) show watermarked video frame and recovered watermark without interlacing, (e) and (f) show watermarked video frame and recovered watermark with one-level interlacing, and (g) and (h) show watermarked video frame and recovered watermark with two-Level interlacing.

**Figure 13:**
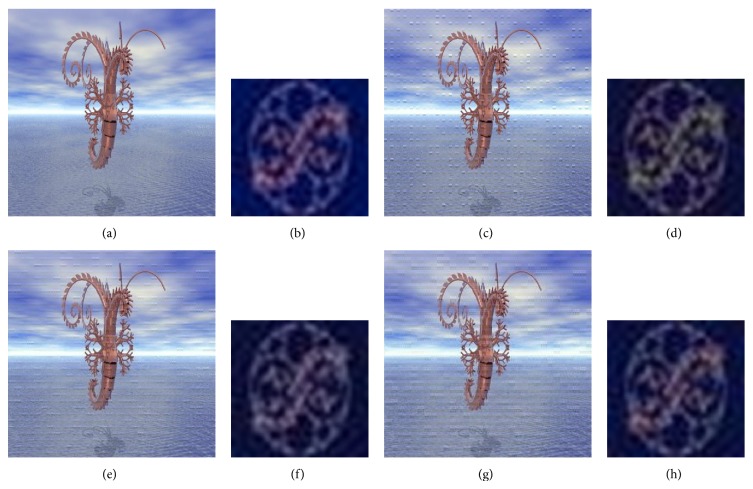
Comparison between the classical non-blind Video Multiple Watermarking System (Video Watermark) and the Proposed Method: where (a) and (b) Original Video Frame and Original Watermark, (c) and (d) Watermarked Video Frame and Recovered Watermark without Interlacing, (e) and (f) Watermarked Video Frame and Recovered Watermark with One Level Interlacing, (g) and (h) Watermarked Video Frame and Recovered Watermark with Two Level Interlacing.

A comparison is made between a classical video multiple watermarking system (without interlacing) and the proposed system (with interlacing). Figures 11, 12, and 13 present this comparison without attacks, one watermark in each figure, and in these figures the comparison is illustrated by showing the watermarked video frame and the extracted watermark before and after applying the proposed method. From these figures it is obviously that there is no degradation in the quality of the original video frame and the original watermark after watermarking using the nonblind watermarking system without interlacing and the same system with interlacing.

Also, Table 2 presents the same comparison for each watermark with the attacks. For image, the watermarking and attacking operations are performed on only one frame, but in the video, the watermarking and attacking operations are performed on a number of frames equal to the number of watermark frames (21 frames), and the values of NC and PSNR are the average values for all color bands and for all watermarked frames as shown in Table 3.

From the results of Table 2, there are three notes as follows.

- First, the results of the watermarking system with and without interlacing are close to each other which indicates that the goal of this paper is achieved without any degradation in its imperceptibly or its robustness against attacks.
- Second, no enhancement in simulation results in two-level interlacing over one level interlacing which indicates that there is no need for more levels of interlacing to save processing time and speed up watermarking process. Also, in the view of code complexity the one-level interlacing is more preferred than the two-level interlacing.
- Third, about the attacks, in general, the results are close to each other between the classical system without interlacing and the same system with interlacing.

These notes are the same notes as [4], which indicates that our proposed solution (image interlacing) is working in all types of nonblind watermarking systems (image single watermarking and video multiple watermarking).

## 6. Conclusion

In this paper, a robust video multiple watermarking technique was proposed to solve nonblind watermarking system problems. This technique was based on image interlacing technique. In this technique three-level discrete wavelet transform (DWT) was used as watermark embedding/extracting domain and Arnold transform as watermark encryption/decryption method. In this paper, different types of media as gray image, color image, and video were used as watermarks. The robustness of this technique was tested by applying different types of attacks. Simulation results showed the effectiveness of the proposed method with all types of nonblind watermarking systems; there is no need to the original host signal in the watermark extraction process and no overhead over system resources. Also, there is no degradation in performance of these systems after applying this solution and no degradation in their robustness against attacks.

Also, simulation results showed the effectiveness and good performance of this proposed technique with saving system resources, memory capacity, and communications bandwidth. The percentage of saving memory and bandwidth is 50% due to prevent sending the original video in the proposed video watermarking system.

## Figures and Tables

**Table 1:** Video interlacing operation.

Interlacing level	Subvideos	NC		
		Red	Green	Blue
One	ER-OR	0.9548	**0.9645**	0.9657
	**EC-OC**	**0.9713**	**0.9760**	**0.9755**

Two	EE-OE	0.9384	**0.9516**	0.9516
	EE-EO	0.9713	**0.9760**	0.9755
	EE-OO	0.9245	**0.9386**	0.9376
	OE-EO	0.9226	**0.9371**	0.9365
	**OE-OO**	**0.9714**	**0.9760**	**0.9756**
	EO-OO	0.9384	**0.9513**	0.9516

Subvideos: ER = even rows, OR = odd rows, EC = even columns, OC = odd columns, EE = even rows even columns, EO = even rows odd columns, OE = odd rows even columns, and OO = odd rows odd columns.

**Table 2:** Multiple Watermarks (Gray Image, Color Image and Video).

	Grayscale image watermark			Color image watermark			Video watermark	
Interlacing level	No	One	Two	No	One	Two	One	Two
Subvideos		EC-OC	OE-OO		EC-OC	OE-OO	EC-OC	OE-OO
Scaling factor (*α*)	0.5	1.8	1.8	0.5	1.8	1.8	4	4
PSNR	36.7755	35.2069	33.9840	36.7755	35.2069	33.9840	28.2955	28.2710
NC								
No attacks	0.9824	0.9578	0.9725	0.9824	0.9578	0.9725	0.9358	0.9486
Cropping 1/8 intermediate	0.4251	0.6129	0.6413	0.4251	0.6129	0.6413	0.7682	0.7800
Cropping 1/8 left corner	0.7250	0.7252	0.7303	0.7250	0.7252	0.7303	0.8029	0.8174
Gaussian noise 0.001	0.8981	0.9438	0.9606	0.8981	0.9438	0.9606	0.8751	0.8911
Gaussian noise 0.01	0.6231	0.8794	0.8962	0.6231	0.8794	0.8962	0.8724	0.8868
Gaussian noise 0.05	0.3181	0.7290	0.7462	0.3181	0.7290	0.7462	0.8579	0.8746
Salt and pepper noise 0.001	0.9603	0.9562	0.9706	0.9603	0.9562	0.9706	0.9343	0.9471
Salt and pepper noise 0.01	0.8100	0.9382	0.9556	0.8100	0.9382	0.9556	0.9199	0.9342
Salt and pepper noise 0.05	0.5351	0.8697	0.8783	0.5351	0.8697	0.8783	0.8630	0.8805
Median filtering	0.8677	0.9507	0.9672	0.8677	0.9507	0.9672	0.9211	0.9341
JPEG compression 70	0.8902	0.9338	0.9473	0.8902	0.9338	0.9473	0.9522	0.9612
JPEG compression 50	0.8734	0.9174	0.9299	0.8734	0.9174	0.9299	0.9462	0.9423
JPEG compression 30	0.8098	0.9070	0.9122	0.8098	0.9070	0.9122	0.9415	0.9448
Brightening	0.5780	0.8869	0.8514	0.5780	0.8869	0.8514	0.8621	0.8477
Darkening	0.6584	0.9101	0.8870	0.6584	0.9101	0.8870	0.8838	0.8762
Sharpening	0.9002	0.8591	0.8708	0.9002	0.8591	0.8708	0.8903	0.8942

**Table 3:** Video Watermark (without interlacing) for all video frames.

Watermark type	Video frames number											
Frame number	1	2	3	4	5	6	7	8	9	10 : 20	21	Average
PSNR	29.1022	29.7661	30.2200	30.2013	30.4375	30.4683	30.5608	30.5818	30.5984	⋯	28.7894	30.1212
NC												
No attacks	0.9575	0.9537	0.9542	0.9587	0.9544	0.9499	0.9486	0.9524	0.9536	⋯	0.9614	0.9543
Cropping 1/8 intermediate	0.9428	0.9312	0.9365	0.9378	0.9327	0.9236	0.9277	0.9247	0.925	⋯	0.9469	0.9314
Cropping 1/8 left corner	0.9262	0.9111	0.913	0.9294	0.9357	0.9152	0.9103	0.9152	0.9224	⋯	0.9278	0.9210
Gaussian noise 0.001	0.8613	0.8395	0.8383	0.8479	0.826	0.815	0.8075	0.8115	0.8085	⋯	0.8824	0.8304
Gaussian noise 0.01	0.869	0.8416	0.8328	0.8454	0.8267	0.8169	0.8071	0.8079	0.8062	⋯	0.8766	0.8309
Gaussian noise 0.05	0.8499	0.8125	0.8146	0.8358	0.8147	0.8003	0.7797	0.7939	0.7851	⋯	0.8601	0.8115
Salt and pepper noise 0.001	0.9559	0.9499	0.9498	0.956	0.9502	0.9455	0.9439	0.9465	0.9492	⋯	0.9594	0.9502
Salt and pepper noise 0.01	0.9326	0.9206	0.9168	0.9274	0.9185	0.9081	0.9052	0.91	0.9071	⋯	0.9405	0.9175
Salt and pepper noise 0.05	0.8515	0.8204	0.8108	0.8288	0.803	0.785	0.7781	0.7779	0.7751	⋯	0.8684	0.8062
Median filtering	0.9173	0.9108	0.9015	0.914	0.9011	0.8867	0.8771	0.8685	0.8818	⋯	0.928	0.8976
JPEG compression 70	0.956	0.9523	0.9522	0.957	0.9532	0.948	0.9467	0.9504	0.9516	⋯	0.9603	0.9526
JPEG compression 50	0.9511	0.945	0.947	0.9521	0.9475	0.9431	0.9397	0.9443	0.9456	⋯	0.9551	0.9467
JPEG compression 30	0.9486	0.9408	0.9424	0.9487	0.9435	0.9383	0.9354	0.9384	0.9406	⋯	0.9531	0.9424
Brightening	0.8696	0.8423	0.8404	0.871	0.8489	0.8226	0.8081	0.7949	0.8031	⋯	0.895	0.8344
Darkening	0.8927	0.8721	0.869	0.8954	0.8767	0.855	0.8421	0.8341	0.842	⋯	0.9138	0.8652
Sharpening	0.9344	0.931	0.9339	0.9376	0.9313	0.9223	0.9243	0.9246	0.9228	⋯	0.9397	0.9294
